# Metal Artifact Reduction Sequences MRI: A Useful Reference for Preoperative Diagnosis and Debridement Planning of Periprosthetic Joint Infection

**DOI:** 10.3390/jcm11154371

**Published:** 2022-07-27

**Authors:** Changyu Huang, Yang Chen, Haiqi Ding, Zida Huang, Chaofan Zhang, Wenbo Li, Xi Liu, Zhanhai Tu, Wenming Zhang, Xinyu Fang

**Affiliations:** 1 Department of Orthopedic Surgery, The First Affiliated Hospital of Fujian Medical University, Fuzhou 350000, China; cyhuang0520@fjmu.edu.cn (C.H.); chenyang0596@163.com (Y.C.); dhq9119@163.com (H.D.); huangzida@163.com (Z.H.); drcfzhang@gmail.com (C.Z.); liwenbo350001@163.com (W.L.); 2 Department of Radiology and Imaging, The First Affiliated Hospital of Fujian Medical University, Fuzhou 350000, China; liuxidehmf@163.com (X.L.); tuzhanhai@163.com (Z.T.)

**Keywords:** periprosthetic joint infection, metal artifact reduction sequences, MRI, diagnosis

## Abstract

The diagnosis and treatment of periprosthetic joint infection (PJI) is complex and the use of MRI in PJI is gaining attention from orthopedic surgeons as MR technology continues to advance. This study aimed to investigate whether metal artefact reduction sequence (MARS) MRI could be used as an adjunct in the preoperative diagnosis of PJI and to explore its role in PJI debridement planning. From January 2020 to November 2021, participants with metal joint prostheses that needed to be judged for infection were prospectively enrolled. According to Musculoskeletal Infection Society standards, 31 cases were classified as infection, and 20 as non-infection. The sensitivity and specificity of MARS MRI for the diagnosis of PJI were 80.65% and 75%, respectively. In MARS MRI, the incidence of bone destruction, lamellar synovitis, and extracapsular soft tissue oedema were significantly higher in PJI than in non-PJI. Fourteen suspicious occult lesions were found in the preoperative MARS MRI in 9 cases, and the location of 9 infection lesions was confirmed intraoperatively. In conclusion, MARS MRI is an effective diagnostic tool for PJIand can provide a visual reference for preoperative surgical planning.

## 1. Introduction

**Figure 1:**
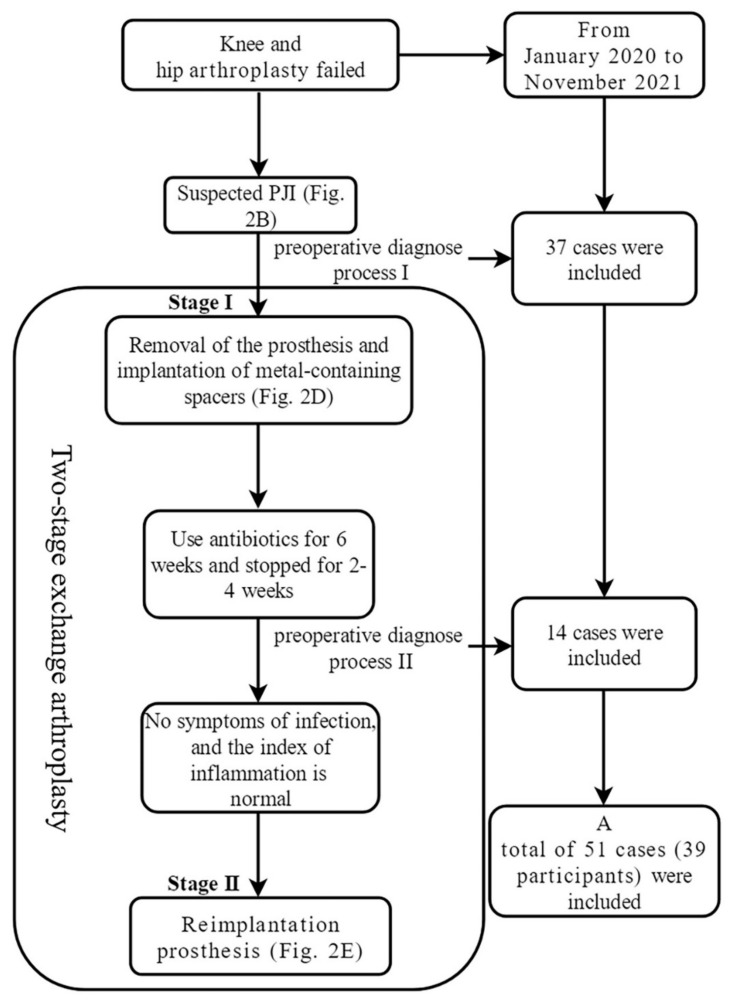
Flow chart of two-stage exchange arthroplasty and inclusion of cases. PJI: periprosthetic joint infection.

**Figure 2:**
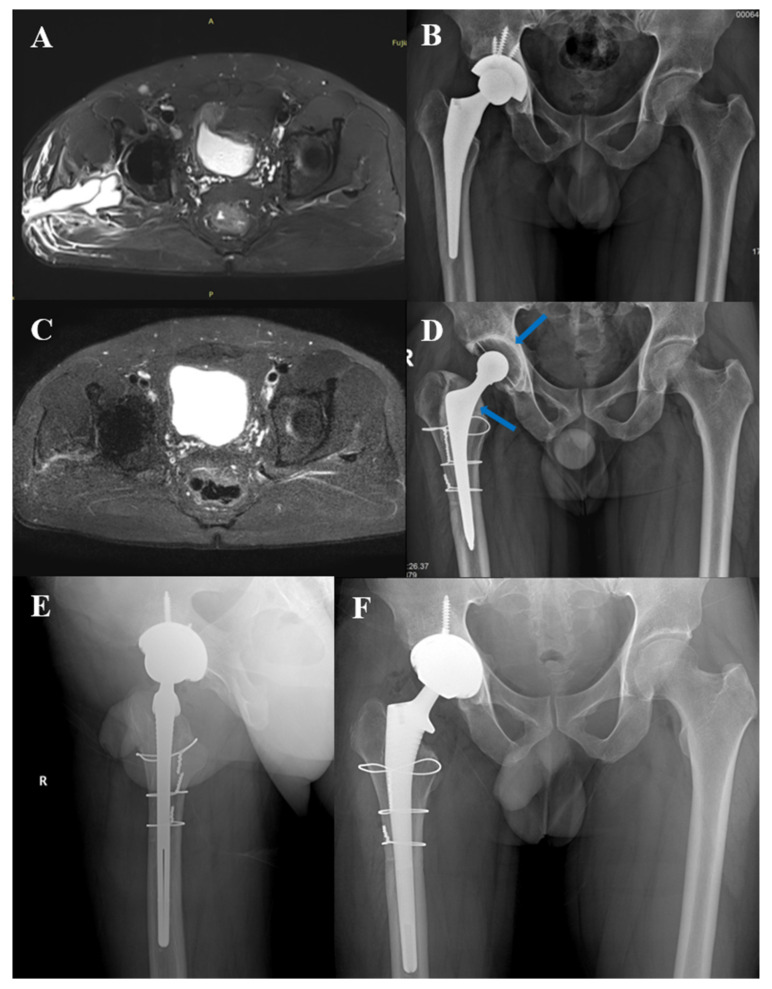
Typical case 1. Male, 41 years old, right total hip arthroplasty. (**A**): MARS MRI before stage I resection surgery; (**B**): X-ray before stage I resection surgery; (**C**): MARS MRI before stage II reimplantation; (**D**): X-ray before stage II reimplantation, the arrow shows bone cement spacer containing metal; (**E**,**F**): X-ray after stage II reimplantation.

Periprosthetic joint infection (PJI) is a serious complication after joint arthroplasty. In particular, for chronic PJI, the current standard treatment is two-stage revision, including stage I prosthesis removal and stage II reimplantation of the prosthesis [1,2]. Its diagnosis and treatment process are complex (Figure 1 and Figure 2).

The Musculoskeletal Infection Society (MSIS) international consensus is effective in the diagnosis of PJI [3,4]; however, there are still many difficulties in the preoperative diagnosis of chronic PJI. Before stage I surgery, it was difficult to determine whether the patient had an infection. Some pathogenic bacteria are low virulence, and inflammatory indicators may be below the threshold of MSIS diagnostic criteria. Additionally, nonstandard pathogen testing may result in a low positive rate of preoperative synovial fluid cultures [5]. It is also difficult to determine whether the infection has been controlled before the stage II surgery. This may be due to the prolonged use of anti-inflammatory drugs and antibiotics, as inflammatory indicators cannot reflect the infection control status. Furthermore, there are also many uncertainties in debridement procedures. In most cases, the surgeon judges the extent of debridement based on the naked eye. If the lesions cannot be distinguished or cannot be found, it may lead to recurrence.

Non-invasive imaging provides useful information to the surgeon prior to surgery. Previously, artifacts caused by metal prostheses limited the application of MR in PJI and guidelines did not recommend it as a diagnostic reference [3,6]. In recent years, many strategies have been effective in reducing metal artifacts of MRI, such as high bandwidth sequences, view angle tilling, multi-acquisition variable resonance image combination, slice encoding for metal art correction, etc. [7,8,9]. They are referred to as metal artifact reduction sequences (MARS). MARS reveals the metal-bone interface, bone, and soft tissue surrounding the prosthesis and makes the preoperative diagnosis of PJI possible. Recently, Galley et al. applied the MARS technique to diagnose PJI after THA with satisfactory results [10]. Our hospital has routinely applied MARS preoperatively for PJI, and we have found that in addition to its value in diagnosis, MARS provides a good reference for PJI debridement planning.

Therefore, we designed this prospective study to investigate the following questions: 1, whether MARS can be used for preoperative diagnosis of PJI and what are the characteristics of MARS MRI of PJI; 2, whether MARS can help in the debridement planning of PJI.

## 2. Materials and Methods

### 2.1. Participant Selection and Clinical Data Collection

This study has been approved by the Ethics Committee of our hospital and all participants signed informed consent forms. Suspected cases of PJI were prospectively included from January 2020 to November 2021. Inclusion criteria: 1, participants who failed hip and knee arthroplasty and were ready for revision surgery; 2, participants diagnosed as chronic PJI and who had undergone stage I resection surgery including metal spacer implantation [11] and planned to undergo stage II reimplantation surgery. Exclusion criteria: 1, incomplete MARS MRI or substandard image quality; 2, incomplete revision surgery. The diagnosis of PJI was determined according to the MSIS criteria [4].

Participants with chronic PJI often undergo two-stage exchange arthroplasty (Figure 1). Each preoperative judgment of infection was an independent and complete diagnosis process (As the case is shown in Figure 2, at the time of initial admission, it is necessary to determine whether there is an infection, which is a diagnostic process. After a confirmed diagnosis of chronic PJI, stage I resection surgery of the two-stage exchange arthroplasty is performed. Three months later, when the case is ready for stage II reimplantation surgery, the status of infection control needed to be determined again). Therefore, we defined each hospitalization of these participants as an independent case.

According to the inclusion criteria, a total of 59 cases were selected and 8 cases were excluded, including 6 cases with incomplete MARS data and 2 cases without surgery. Finally, 51 cases were included in the study, and data on X-ray, ESR, CRP, synovial fluid white blood cells (SF-WBCs) and percentage of polymorphonuclear leukocytes (SF-PMN%), etc., were also collected.

### 2.2. MARS Protocol

MARS MRI was performed more than 8 weeks after the last procedure. MRI examinations were performed using a 3.0-T device (Skyra, Siemens Healthcare, Germany) by the body-flexed array coil with participants in the supine position. The MARS protocol is listed in Table 1. Some parameters were slightly changed as needed.

### 2.3. Definition of MARS Characteristic Performance on PJI

**Figure 3:**
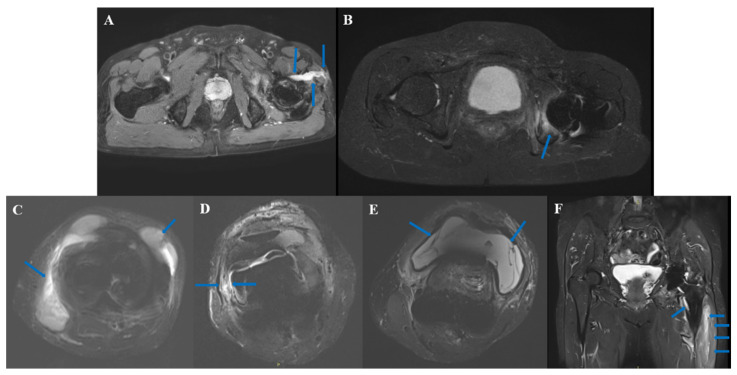
MARS characteristic performance on PJI. (**A**): sinus; (**B**): bone edema; (**C**): extracapsular collections; (**D**): lamellated synovitis; (**E**): intracapsular collections; (**F**): bone destruction: arrow at the lesser trochanter; extracapsular soft-tissue edema: juxtaposed arrow.

According to previous reports [12,13], the characteristics of PJI on MARS are as follows: sinus tract (Figure 3A): latent canal extending to the skin, communicating with joints, signal intensity is higher than surrounding soft tissue in T1; bone edema (Figure 3B): higher signal intensity of bone on T1; extracapsular collections (Figure 3C): extracapsular fluid signal; lamellated synovitis (Figure 3D): composed of multi-layer synovium, synovium shows high T1 signal in contrast to skeletal muscle [14]; intracapsular collections (Figure 3E): intracapsular fluid signal; bone destruction (Figure 3F, arrow at the lesser trochanter): bone defect with higher T1 signal; extracapsular soft-tissue edema (Figure 3F, juxtaposed arrow): T1 higher signal intensity of the surrounding tissue outside the articular.

### 2.4. MRI Analysis

The MRI was evaluated by an orthopedic surgeon with more than 30 years of experience and a musculoskeletal radiologist with more than 20 years of experience. The participant information of the MRI was not provided. They judged whether there was sinus, bone edema, extracapsular collections, lamellated synovitis, intracapsular collections, or bone destruction, and extracapsular soft-tissue edema on the MRI was then made. Then, they judged whether there was PJI. When there was a disagreement, the two evaluators reached a consensus on diagnosis through consultation. One month after the initial assessment, the MRI order of the cases was disrupted, and then they conducted a PJI assessment again to determine the intra-observer reliability. The analysis of diagnostic effectiveness was based on the data obtained from the first evaluation.

### 2.5. Definition of Suspicious Occult Infectious Lesions

In this study, suspicious occult infectious lesions were defined as bone destruction or pus accumulation that are difficult to detect during conventional surgery (hip: posterolateral approach, knee: medial parapatellar approach). Three types of lesions were selected that would significantly affect the operation plan, including the lesions in the bone marrow, the back of the acetabular prosthesis, and the encapsulated lesions in the soft tissue. Before surgery, the surgeon examined the MRI and recorded when the above lesions were present; the corresponding site was explored during the operation. The definition of the infection site during the operation is a purulent focus with the naked eye, and the histopathology of the site is positive according to the MSIS standard [4].

### 2.6. Statistical Analysis

Variables with normal distribution are described by mean ±standard deviation, and variables with non-normal distribution are described by median and inter-quartile ranges. According to the characteristics of the variables, *t*-test, chi-square test, Fisher’s exact test, or the Mann-Whitney test were used to analyze the statistical differences. The intra-observer reliability was analyzed by κ-test. McNemar’s test was used to analyze the sensitivity and specificity of MARS MRI and culture, CRP, ESR, SF-WBC, and SF-PMN%. All *p* values reported were results from two-sided tests, and when *p* < 0.05, they were considered statistically significant. SPSS 26.0 (IBM, Armonk, NY, USA) was used for statistical analysis.

## 3. Results

### 3.1. Demographics of Cases

A total of 51 cases (39 participants) with an average age of 60.57 years (from 32 to 88 years old) were included, including 34 females, 17 males, 30 hips and 21 knees. According to the MSIS standard, there were 31 cases classified as PJI, including 21 cases of mono-pathogen infection, 2 cases of multi-pathogen infection and 8 cases of culture-negative PJI. All microbiology data are presented in Table 2. A total of 20 cases were classified as non-PJI, including 14 cases undergoing stage II reimplantation of two-stage revision and postoperative confirmation of infection control (two cases had a positive intraoperative culture from a single specimen, considered as specimen contamination; postoperative follow-up has not shown recurrence of infection up to now, with follow-up times of 9 and 12 months, respectively), and 6 cases with aseptic loosening underwent one-stage revision. The clinical data are shown in Table 3.

### 3.2. MARS MRI Features of PJI and its Diagnostic Efficiency

Among MARS MRI features, PJI cases had higher probabilities of bone destruction (*p* < 0.05), lamellar synovitis (*p* = 0.01), and extracapsular soft-tissue edema (*p* < 0.001) than non-PJI cases. There was no significant difference in sinus (*p* = 0.15), bone edema (*p* = 0.13), intracapsular collections (*p* = 0.74), extracapsular collections (*p* = 0.54). Sinus had the highest specificity (100%) with a positive predictive value of 100% and a negative predictive value of 42.55%. Extracapsular edema had the highest sensitivity (90.32%) with a positive predictive value of 80.00% and a negative predictive value of 81.25%. The relevant data are presented in Table 4.

### 3.3. Diagnostic Efficacy of MARS MRI

The κ-test was performed on the two diagnoses made by the evaluators, and the κ-value was 0.70 (95%CI 0.50,0.90), *p* < 0.001, which considered the evaluation to be robust.

The sensitivity of MARS MRI for the diagnosis of PJI was 80.65%, which was not statistically different with culture, CRP, ESR, SF-WBCs, and SF-PMN%; the specificity was 75%, which was not statistically different with culture, CRP, ESR, and SF-WBC; Youden’s index was 0.56, which was lower than culture (0.64), CRP (0.74), SF-WBCs (0.70), SF-PMNs% (0.61), and higher than ESR (0.41).

After selecting three characteristic expressions of bone destruction, lamellated synovitis, and extracapsular soft-tissue edema for pairwise paralleling, the sensitivity of lamellated synovitis and extracapsular soft-tissue edema in parallel increased to 93.59%, and negative predictive value increased to 83.33%. Relevant data are listed in Table 5.

### 3.4. Suspicious Occult Infectious Lesions Revealed by MARS MRI

According to the definition described in the methodology, MARS MRIs suggested that the suspicious occult infectious lesions might be present in 9 PJI cases (14 sites in total). Finally, 9 sites were confirmed as infectious lesions by intraoperative exploration and pathology. Including (lesions detected by intraoperative exploration/lesions detected by MRI before operation): bone marrow 3/6, back of the acetabular prosthesis 3/5, and encapsulated lesions in the soft tissue 3/3. These suspicious occult lesions found in MRIs led to the change of surgical plan for the expanding scope of debridement, including extended trochanteric osteotomy (2 cases), tibial tubercle osteotomy (1 case), acetabular revision (3 cases), and extended incision (2 cases).

### 3.5. Typical Cases

Case in Figure 2: Male, 41 years old, one year after prime hip arthroplasty, with a sinus found in the hip for two weeks. At the MRI before stage I resection surgery, a sinus tract was visible, as was extensive edema of the soft tissue around the prosthesis (Figure 2A). The MRI after 10 months of surgery showed that the extracapsular edema had disappeared (Figure 2C), and stage II reimplantation was performed.

**Figure 4:**
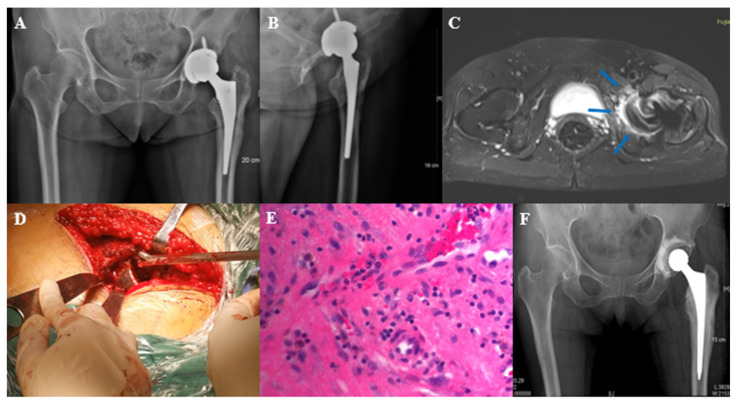
Typical case 2. Female, 68 years old, left total hip arthroplasty. (**A**,**B**): X-ray before stage I resection surgery; (**C**): MARS MRI showed signs of acetabular bone destruction and bone edema (at the arrow); (**D**): intraoperative exploration revealed occult lesions in the acetabulum; (**E**): histopathology of the acetabulum suggests infection; (**F**): X-ray after stage I resection surgery.

Case in Figure 4: Female, 68 years old, two years after prime hip arthroplasty, with hip pain and swelling for one month. The patient’s main symptom before revision was pain in the left thigh. Therefore, the initial surgical plan was to perform a femoral side revision. Preoperative MARS MRI revealed occult lesions (bone destruction and edema) on the dorsal side of the acetabular cup (Figure 4C), suggesting the presence of infection. Although the acetabular cup was found to be firmly fixed during revision surgery, we removed it. Infection was found during the revision of the acetabular side (Figure 4D), and the histopathology of the acetabular side during the operation showed that there were more than 5 neutrophils in the high magnification field (Figure 4E). Culture results: *Methicillin sensitive staphylococcus aureus.*

## 4. Discussion

In patients with suspected PJI, preoperative confirmation of infection is important. In addition to collecting blood inflammatory indicators, conventional detection methods including ultrasound-guided puncture, optimizing culture flow, and next-generation sequencing have been reported to improve the preoperative diagnostic efficacy [15,16,17]. We used the above methods. However, only 64.52% (20/31) of the cases of infection finally diagnosed in this study met the MSIS criteria preoperatively.

Although Galley’s retrospective studies on THA have demonstrated the superiority of MARS [10], whether it is still effective as a diagnostic tool in cases where the preoperative infection is unclear, therefore, we designed this prospective study. In this study, the sensitivity of MARS MRI for the preoperative diagnosis of PJI was 80.65% and the specificity was 75%, which was not a statistically significant difference compared with conventional diagnostic indices (culture, CRP, ESR, SF WBCs, SF PMNs%) (*p* > 0.05), indicating that the diagnostic efficacy of MARS MRI is not inferior to the commonly used diagnostic indicators. Moreover, compared with the preoperative culture, the diagnostic accuracy of MARS is better (Youden’s index:0.173, 0.556, respectively), and it also has the advantage of being noninvasive and easily acquired preoperatively.

We introduced commonly used MRI judgment indicators of musculoskeletal infection including bone edema, extracapsular collections, lamellated synovitis, intracapsular collections, bone destruction, and extracapsular soft-tissue edema [12,13]. The occurrence rate of bone destruction, lamellated synovitis, and extracapsular soft-tissue edema in PJI were significantly higher than that of non-PJI, indicating that these indexes may be the PJI characteristics in MARS MRI. Plodkowski et al. reported that lamellated synovitis has a sensitivity of 92% and a specificity of 85% in the diagnosis of knee PJI [14], which is higher than our results (64.52%, 75%, respectively). This may be due to the introduction of hip joint cases in this study. In our study, extracapsular soft-tissue edema is sensitive and easy to identify on MRI (sensitivity 90.32%), which is similar to that reported by Galley (95%) [10]. However, the extracapsular soft-tissue edema in 3 cases of PJI in this study was judged to be negative before operation. The cases were all knee PJI, whose MRI still had relatively obvious artifacts, and although we adjusted the scanning parameters, it was still difficult to avoid artifacts. This may be due to the complex geometry of the knee prosthesis, which may also account for the lower specificity (65%) in our study than in Galley’s study (86%) [10].

To improve sensitivity, lamellar synovitis and extracapsular, soft-tissue edema were selected as pairwise parallel tests. The sensitivity reached 93.55% and the negative predictive value was 83.33%. This could serve as a better identification for PJI cases with normal preoperative inflammatory indicators. If the parallel test is negative (Figure 2C), it can assist the surgeon in determining whether the infection has been controlled in patients preparing for stage II reimplantation. There were 2 cases with a positive culture of a single specimen in stage II reimplantation surgery, whose preoperative MARS MRI showed that lamellar synovitis and extracapsular soft-tissue edema outside the joint were negative. Combined with other intraoperative indicators, it was judged as non-PJI according to MSIS criteria and did not prolong antibiotic use. There was no recurrence of infection after follow-up for 9 months and 12 months.

Another advantage of the MARS MRI is that it provides visual images before the operation. We found 14 suspicious occult lesions in the MRI of 9 cases, 9 of which were all confirmed in the same location during the operation. In the case shown in Figure 4, although the main symptom was on the femoral side, we revised the acetabular side according to MRI (Figure 4C) and found the lesion (Figure 4D) corresponding to that shown by MRI. X-rays are valid for post-arthroplasty evaluation [18], and the X-ray, in this case, suggested the periosteal reaction on the acetabular side (Figure 4A,B). However, the MRI sign is more obvious and accurate than the X-ray.

This study also has limitations:

1. Our department had a relatively high percentage of patients with PJI, which inevitably would have biased the selection of cases.

2. Due to the composition of some early implants which cannot be traced, this study did not provide the composition of the prosthesis, including the percentage of cobalt-chromium.

## 5. Conclusions

In conclusion, the diagnostic accuracy of PJI is high when the MARS MRI of patients with suspected PJI shows signs of bone destruction, lamellar synovitis and extracapsular soft-tissue edema. We can accurately determine that the infection has been controlled when MARS MRI shows no lamellar synovitis and extracapsular soft-tissue edema prior to stage II reimplantation surgery. The diagnostic efficacy of MARS MRI for PJI preoperatively is not inferior to that of conventional diagnostic indices. It improves the efficiency of debridement by providing a visual reference for surgical planning preoperatively, which will make debridement more thorough and avoid overlooking occult infectious lesions.

## Figures and Tables

**Table 1:** Protocol of metal artifact reduction sequence.

		TE (ms)	TR (ms)	TI (ms)	FOV (mm)	NEX	RBW (kHZ)	ST (mm)	Matrix	VAT	Flip Angle (Degrees)
Hip	T1w tse Tra	7.7	620		360 × 360	1	625	4	320 × 192	On	144
	PD tirm Cor	36	4160	220	340 × 340	2	601	4	320 × 256	On	138
	PD tirm Tra	36	3500	220	360 × 270	2	601	4	320 × 256	On	137
Knee	T1w tse Sag	8.7	420		180 × 180	1	625	4	320 × 224	On	137
	PD tirm Sag	33	5380	220	180 × 180	2	601	4	320 × 192	On	138
	PD tirm Cor	33	5380	220	180 × 180	2	601	4	320 × 192	On	138
	PD tirm Tra	33	3500	220	200 × 162	2	601	4	320 × 256	On	137

TE: Echo time, TR: Repetition time, TI: Inversion time, FOV: Field of view, NEX: Number of excitations, RBW: Receiver bandwidth, ST: Slice thickness, VAT: View-angle tilting.

**Table 2:** Microbiology finding of culture.

PJI, *n* = 31			Non-PJI, *n* = 20	
Mono-Pathogen, *n* = 21	Multi-Pathogen, *n* = 2	CN PJI, *n* = 8	Aseptic Loosening, *n* = 6	Stage II Reimplantation Surgery, *n* = 14
*Methicillin sensitive staphylococcus aureus*, *n* = 3	*Stenotrophomonas maltophilia, Pseudomonas monteilii, Enterococcus faecalis*, *n* = 1			*Staphylococcus hominis*, *n* = 1
*Klebsiella* *peneumoniae*, *n* = 1	*Ralstonia pickettii*, *Staphylococcus epidermidis*, *n* = 1			*Staphylococcus haemolyticus*, *n* = 1
*Escherichia coli*, *n* = 1				
*Methicillin-resistant staphylococcus aureus*, *n* = 5				
*Aggregatibacter aphrophilus*, *n* = 1				
*Methicillin sensitive staphylococcus* *epidermidis*, *n* = 2				
*Enterobacter cloacae*, n = 1				
*Staphylococcus* *warneri*, *n* = 1				
*Staphylococcus epidermidis*, *n* = 2				
*Pseudomonas* *aeruginosa*, *n* = 1				
*Streptococcus* *hemolyticus*, *n* = 1				
*Candida albicans*, *n* = 2				

PJI: Prosthetic joint infection, CN PJI: Culture-negative prosthetic joint infection.

**Table 3:** Characteristics of cases.

Characteristic		All Cases	PJI	Non-PJI	*p*-Value (PJI vs. Non-PJI)
Female (*n*)		34	18	16	0.105 ^a^
Age (yrs)		60.569 ± 12.400	60.774 ± 13.266	60.250 ± 11.252	0.885 ^b^
Joint involved (*n*)					0.891 ^a^
	hip	30	18	12	
	knee	21	13	8	
CRP (mg/L), median, IQR		11.020 (4.210, 27.260)	19.29 (11.100, 69.300)	4.380 (2.120, 5.823)	<0.001 ^c^
ESR (mm/h), $\bar{X}$ ± S		47.800 ± 33.829	64.650 ± 33.602	28.700 ± 17.147	<0.001 ^b^
SF-WBC (×10^6^/L), median, IQR		2623.000 (795.000, 8024.000)	6185.000 (2623.000, 35,883.000)	672.000 (278.250, 1110.750)	<0.001 ^c^
SF-PMN% (%), median, IQR		66.600 (45.200, 83.900)	81.900 (66.900, 88.100)	45.200 (39.025, 51.225)	<0.001 ^c^

^a^ Chi-squared, ^b^ Independent-samples *t*-test, ^c^ Mann-Whitney U test; PJI: Prosthetic joint infection, SF-WBC: Synovial fluid white blood cell, SF-PMN: Synovial fluid polymorphonuclear, IQR: Interquartile range, CRP: C-reactive protein, ESR: Erythrocyte sedimentation rate.

**Table 4:** Statistical analysis of diagnostic efficiency of each characteristic of MARS MRI in PJI.

Variable	PJI	Non-PJI	*p*- Value ^a^	Sensitivity	Specificity	Youden’s Index	PPV	NPV	LR+	LR−
Sinus	4/31	0/20	0.145	12.90%	100%	0.129	100%	42.55%	N/A	0.871
Bone destruction	17/31	5/20	0.046	54.84%	75%	0.298	77.27%	51.72%	2.194	0.602
Bone edema	23/31	10/20	0.132	74.19%	50%	0.242	69.70%	55.56%	1.484	0.516
Lamellated synovitis	20/31	5/20	0.009	64.52%	75%	0.395	80.00%	57.69%	2.581	0.473
Extracapsular edema	28/31	7/20	<0.001	90.32%	65%	0.7	80.00%	81.25%	2.581	0.149
Intracapsular collections	24/31	14/20	0.743	77.42%	30%	0.074	63.16%	46.15%	1.106	0.753
Extracapsular collections	11/31	5/20	0.543	35.48%	75%	0.105	68.75%	42.86%	1.419	0.860

^a^ Fisher’s exact test; Youden’ s index = sensitivity + specificity − 1; PJI: Prosthetic joint infection, LR+: Positive likelihood ratio, LR**−**: Negative likelihood ratio, NPV: Negative predictive value, PPV: Positive predictive value, N/A: Not applicable.

**Table 5:** Statistical analysis of diagnostic efficiency of each index of MSIS and MARS MRI in PJI.

Variable	PJI	Non-PJI	Sensitivity	*p*-Value ^a^ (VS. MARS)	Specificity	*p*-Value ^a^ (VS. MARS)	Youden’s Index	PPV	NPV	LR+	LR−
MARS MRI	25/31	5/20	80.65%	N/A	75%	N/A	0.556	83.33%	71.43%	3.226	0.258
Culture	23/31	5/20	74.19%	0.727	75%	1.000	0.642	92%	69.23%	7.419	0.287
Pre-OP culture	10/31	3/20	32.26%	0.001	85%	0.625	0.173	76.92%	44.74%	2.151	0.797
Intra-OP culture	21/31	2/20	67.74%	0.344	90%	0.453	0.577	91.30%	64.29%	6.774	0.358
CRP (>10 mg/L)	26/31	2/20	83.87%	1.000	90%	0.375	0.739	92.86%	78.26%	8.387	0.179
ESR (>30 mm/h)	25/31	8/20	80.65%	1.000	60%	0.180	0.406	75.76%	66.67%	2.016	0.323
SF-WBC (>3000 × 10^6^/L)	23/31	1/20	74.19%	0.687	95%	0.125	0.692	95.83%	70.37%	14.84	0.272
SF-PMN% (>80%)	19/31	0/20	61.30%	0.109	100%	N/A	0.613	100%	62.50%	N/A	0.387
Bone destruction+ Lamellated synovitis	26/31	7/20	83.87%	-	65%	-	0.489	78.79%	72.22%	2.396	0.248
Bone destruction+ Extracapsular edema	28/31	9/20	90.32%	-	55%	-	0.453	75.68%	78.57%	2.007	0.176
Lamellated synovitis+ Extracapsular edema	29/31	10/20	93.55%	-	50%	-	0.435	74.36%	83.33%	1.871	0.129

^a^ McNemar’s test; Youden’ s index = sensitivity + specificity − 1; MARS: Metal artifact reduction sequence, PJI: Prosthetic joint infection, LR+: Positive likelihood ratio, LR−: Negative likelihood ratio, NPV: Negative predictive value, PPV: Positive predictive value, N/A: Not applicable, SF-WBC: Synovial fluid white blood cell, SF-PMN: Synovial fluid polymorphonuclear, OP: operative.

## Data Availability

Data will be made available on request.

## References

[1] Kapadia B.H., Berg R.A., Daley J.A., Fritz J., Bhave A., Mont M.A. (2016). Periprosthetic joint infection. Lancet.

[2] Tande A.J., Gomez-Urena E.O., Berbari E.F., Osmon D.R. (2017). Management of Prosthetic Joint Infection. Infect. Dis. Clin. N. Am..

[3] Parvizi J., Gehrke T., Chen A.F. (2013). Proceedings of the International Consensus on Periprosthetic Joint Infection. Bone Jt. J..

[4] Parvizi J., Tan T.L., Goswami K., Higuera C., Della Valle C., Chen A.F., Shohat N. (2018). The 2018 Definition of Periprosthetic Hip and Knee Infection: An Evidence-Based and Validated Criteria. J. Arthroplast..

[5] Karim M.A., Andrawis J., Bengoa F., Bracho C., Compagnoni R., Cross M., Danoff J., Valle C.J.D., Foguet P., Fraguas T. (2019). Hip and Knee Section, Diagnosis, Algorithm: Proceedings of International Consensus on Orthopedic Infections. J. Arthroplast..

[6] Tubb C.C., Polkowksi G.G., Krause B. (2020). Diagnosis and Prevention of Periprosthetic Joint Infections. J. Am. Acad. Orthop. Surg..

[7] Chen C.A., Chen W., Goodman S.B., Hargreaves B.A., Koch K.M., Lu W., Brau A.C., Draper C.E., Delp S.L., Gold G.E. (2011). New MR imaging methods for metallic implants in the knee: Artifact correction and clinical impact. J. Magn. Reson Imaging.

[8] Jungmann P.M., Agten C.A., Pfirrmann C.W., Sutter R. (2017). Advances in MRI around metal: MRI Around Metal. J. Magn. Reson Imaging.

[9] Khodarahmi I., Nittka M., Fritz J. (2017). Leaps in Technology: Advanced MR Imaging after Total Hip Arthroplasty. Semin. Musculoskelet Radiol..

[10] Galley J., Sutter R., Stern C., Filli L., Rahm S., Pfirrmann C.W.A. (2020). Diagnosis of Periprosthetic Hip Joint Infection Using MRI with Metal Artifact Reduction at 1.5 T. Radiology.

[11] Cai Y., Fang X., Huang C., Li Z., Huang Z., Zhang C., Li W., Zhang Z., Guan Z., Zhang W. (2021). Destination Joint Spacers: A Similar Infection-Relief Rate But Higher Complication Rate Compared with Two-Stage Revision. Orthop Surg..

[12] Fritz J., Lurie B., Miller T.T., Potter H.G. (2014). MR Imaging of Hip Arthroplasty Implants. RadioGraphics.

[13] Fritz J., Lurie B., Potter H.G. (2015). MR Imaging of Knee Arthroplasty Implants. RadioGraphics.

[14] Plodkowski A.J., Hayter C.L., Miller T.T., Nguyen J.T., Potter H.G. (2013). Lamellated Hyperintense Synovitis: Potential MR Imaging Sign of an Infected Knee Arthroplasty. Radiology.

[15] Fang X., Zhang L., Cai Y., Huang Z., Li W., Zhang C., Yang B., Lin J., Wahl P., Zhang W. (2021). Effects of different tissue specimen pretreatment methods on microbial culture results in the diagnosis of periprosthetic joint infection. Bone Jt. Res..

[16] Huang Z., Li W., Lee G.-C., Fang X., Xing L., Yang B., Lin J., Zhang W. (2020). Metagenomic next-generation sequencing of synovial fluid demonstrates high accuracy in prosthetic joint infection diagnostics: mNGS for diagnosing PJI. Bone Jt. Res..

[17] Sdao S., Orlandi D., Aliprandi A., Lacelli F., Sconfienza L.M., Randelli F., Sardanelli F., Serafini G. (2015). The role of ultrasonography in the assessment of peri-prosthetic hip complications. J. Ultrasound..

[18] Pozzuoli A., Berizzi A., Crimì A., Belluzzi E., Frigo A.C., Conti G.D., Nicolli A., Trevisan A., Biz C., Ruggieri P. (2020). Metal Ion Release, Clinical and Radiological Outcomes in Large Diameter Metal-on-Metal Total Hip Arthroplasty at Long-Term Follow-Up. Diagnostics.

